# Income gradient of pharmaceutical panic buying at the outbreak of the COVID‐19 pandemic

**DOI:** 10.1002/hec.4378

**Published:** 2021-07-03

**Authors:** Péter Elek, Anikó Bíró, Petra Fadgyas‐Freyler

**Affiliations:** ^1^ Centre for Economic and Regional Studies Budapest Hungary; ^2^ Corvinus University of Budapest Budapest Hungary; ^3^ National Health Insurance Fund Administration Budapest Hungary

**Keywords:** COVID‐19, inequality, panic buying, pharmaceutical demand

## Abstract

We analyze the timing, magnitude, and income dependence of pharmaceutical panic buying around the outbreak of the COVID‐19 pandemic in Hungary. We use district‐level monthly and daily administrative data on detailed categories of pharmaceutical purchases, merge them to income statistics, and estimate multilevel panel models. Our main results are as follows. First, the days of therapy (DOT) of pharmaceutical purchases increased by more than 30% in March 2020, when major lockdown measures were announced. This pattern holds for almost all categories of pharmaceuticals. Second, shortly after the panic reactions, the aggregate amount of pharmaceutical purchases returned to their preshock levels; however, the frequency of pharmacy visits decreased. Third, the panic buying reaction was significantly stronger in richer geographical areas, where—according to the daily data—people also reacted earlier to the pandemic‐related news. Overall, the results suggest that panic buying of pharmaceuticals can have detrimental effects on vulnerable populations.

## 1 INTRODUCTION

The rapid spread of the COVID‐19 disease in the first months of 2020 caused high levels of anxiety in societies and hence resulted in panic buying, that is in hoarding of basic necessities including pharmaceuticals. Panic buying can be a rational reaction because potential supply disruptions, the anticipated restriction of movement, and the risk of disease transmission during store visits all have the effect of increasing optimal inventory holdings. Also, a crisis might lead to higher future prices, increasing current demand. However, the phenomenon of panic buying is socially costly because it can lead to shortages and thus heighten the anxiety about the pandemic (Keane & Neal, 2021). Shortages are especially costly for the vulnerable for whom shopping can be challenging, hence policy interventions may be necessary to address the detrimental impact of panic buying on them (Besson, 2020).

A growing body of the literature uses high‐frequency transaction data to analyze the impact of the COVID‐19 pandemic on consumer spending (Baker et al., 2020; Carvalho et al., 2020; Chetty et al., 2020; O'Connell et al., 2020, among many others) but there is less large‐scale empirical evidence on the impact of the pandemic on pharmaceutical purchases. The available literature suggests that while the outbreak of the pandemic lead to a dramatic decrease in the utilization of outpatient healthcare services (Ahn et al., 2020; Cantor et al., 2020; Chatterji & Li, 2021; Ziedan et al., 2020), there was also a temporary surge in the purchases in pharmaceuticals. Using weekly wholesale data from Germany, Kostev and Lauterbach (2020) show evidence for a significant surge in purchases of medications for various chronic diseases shortly prior to the COVID‐19 lockdown. Clement et al. (2021) document a surge in the demand for prescription drugs in March 2020 in the United States and also prove that the likelihood of discontinuing some medications increased and the number of new patients decreased after the spread of COVID‐19. Our main contributions to this evolving literature are twofold. First, we estimate the exact timing and magnitude of panic buying of all categories of pharmaceuticals using administrative data of monthly and daily frequency from Hungary. Second, by observing the district of the patients, we investigate the socioeconomic differences in the patterns of pharmaceutical panic buying. While our focus is on the impacts of the COVID‐19 shock, the results have a broader relevance—Loxton et al. (2020) document that consumer behavior during the COVID‐19 crisis appears to align with behaviors exhibited during historic shock events.

## 2 BACKGROUND

### 2.1 Milestones

**FIGURE 1:**
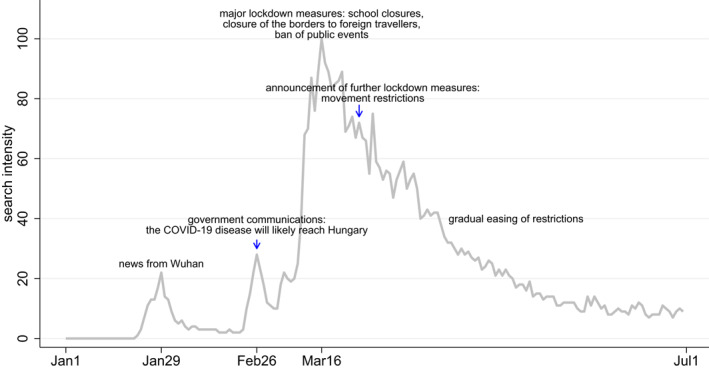
Google search intensity for “coronavirus” or “covid” in Hungary. *Source*: Google trends. Time period: January 1, 2020–June 30, 2020. The search intensity indicator is set to 100 at its peak in the analyzed time period

In the first half of 2020, Hungary was moderately affected by the COVID‐19 pandemic. The first COVID‐19 cases were registered on March 5, 2020, the first death occurred on March 16, 2020. Until June 30, 2020, there were 4145 cases and 585 deaths (out of the population of 9.8 million) (WHO, 2020). However, the rising numbers in nearby countries were perceived as a major threat for Hungary around the end of February 2020, and this was reflected by government communication and by the rising Google search intensity for the term “coronavirus” (“koronavírus,” in Hungarian) or “covid” at that time (Figure 1). On March 11, 2020, the Government declared state of emergency, banned large gatherings and ordered the closure of universities. On March 13, 2020, the Prime Minister announced the closure of schools as of March 16, 2020. Further lockdown measures were implemented on March 16, 2020, including the closure of the borders to foreign travelers and the ban of all public events. Movement restrictions were introduced as of March 28, 2020: individuals were allowed to leave their homes only for essential needs, exercise, and work‐related reasons. The restrictions were gradually eased from the end of April 2020, and the state of emergency was lifted on June 17, 2020.

### 2.2 Institutional background

In Hungary, user fees for prescription medications depend on the subsidy rates from the social security, which vary between 25% and 100%, and are slightly less than 50% on average. To get a prescription, patients have to contact a physician (typically the primary care physician) either at a clinic or by phone. Outpatient and inpatient healthcare visits do not require copayments. Physicians are allowed to provide prescription for at most 3‐month supply for patients with chronic conditions and for 1 month otherwise (Gaál et al., 2011 provide a detailed overview of the Hungarian healthcare system.).

## 3 DATA

The prescription drug data originate from the National Health Insurance Fund Administration, the single payer of the Hungarian healthcare system. The data are on the level of the 197 districts of Hungary (LAU1—local administrative unit level 1), with an average population of about 50,000 people.

First, we have monthly information on the district‐level days of therapy (DOT) as well as the number of patients who bought medication for each first level ATC (Anatomical Therapeutic Chemical) group, and specifically for antidiabetics (ATC A10), antihypertensives (ATC C02–C03, C07–C09) and antidepressants (ATC N06A). In the analysis, we use per capita values, after adjustment to the average gender and age distribution of Hungary. Time coverage is January 2017–July 2020.

Second, we have district‐level daily data on per capita DOT of antidiabetics, antihypertensives, and antidepressants. Time coverage is January 1, 2020–June 30, 2020.

We merge the dataset to the year 2017 values of district‐level annual per capita taxable income, which originate from the National Regional Development and Spatial Planning Information System (TeIR).

## 4 METHODS

First, we model log *y*_*it*_, the logarithm of gender‐ and age‐adjusted per capita *monthly* consumption (DOT or number of patients) of a drug category in district *i* in month *t* (running from January 2017 until July 2020) as follows:

(1) $$\log y_{it}=\alpha_{q}t+\beta_{q}w_{t}+\overset{12}{\underset{j=1}{\sum }}\gamma_{qj}m_{jt}+\overset{7}{\underset{k=1}{\sum }}\delta_{qk}m_{2020k,t}+p_{i}+u_{t}+\varepsilon_{it},$$

where *t* is the time trend, *w*_*t*_ is the number of working days in a month, *m*_*jt*_ is the dummy for calendar month *j*(*j* = 1, 2, …, 12), *m*_2020*k*,*t*_ is the dummy for month *k* in year 2020 (*k* = 1, 2, , …, 7 due to the date range), and *p*_*i*_ is the district fixed effect. Note that all parameters (*α*_*q*_, *β*_*q*_, *γ*_*qj*_, *δ*_*qk*_) are specific to the income tertile of the district (indexed by *q* = 1, 2, 3). The parameters of interest are *δ*_*qk*_, which show the income‐dependent deviation of pharmaceutical purchases in the first 7 months of 2020 from the trend and seasonality of the preceding 3 years.

Since the monthly shocks are correlated across districts, we model the (composite) error term as *u*_*t*_ + *ɛ*_*it*_, where *u*_*t*_ is a random month effect, *ɛ*_*it*_ is the residual, and they are zero‐mean, normally distributed, serially uncorrelated random variables, also independent from each other. The model is estimated with maximum likelihood, using the *mixed* command of the Stata software package.

Second, for the *daily* data, let *i* denote the district and *t* the working days (we exclude purchases on weekends and national holidays, which altogether make up around 5% of total consumption.) Since the daily data only cover year 2020, we need to model intramonthly patterns in order to find the unusual days when purchases suddenly increased. As Figure 5 shows, purchases are highly seasonal within a month, and reach their maximum on the 12th day (or on the last working day before), which is the time of the payment of pensions and other pension‐type benefits in Hungary. Hence, we model log *y*_*it*_ as follows:

(2) $$\log y_{it}=\sum_{j=-9}^{9}\left(\theta_{j0}+\theta_{j1}s_{i}\right)d_{jt}+\sum_{k=1}^{5}\kappa_{k}f_{jt}+\lambda_{-1}g_{-1,t}+\lambda_{+1}g_{+1,t}+p_{i}+u_{0t}+u_{1t}s_{i}+\varepsilon_{it},$$

where *d*_*jt*_ is the dummy variable indicating the *j*th working day (−9 ≤ *j* ≤ 9) relative to the above defined peak day of drug purchases within a month, *f*_*jt*_ indicates within‐week seasonality (*j* = 1, 2, …, 5), while *g*_−1,*t*_ and *g*_+1,*t*_ denote the working days before and after a national holiday, respectively, and *p*_*i*_ is the district fixed effect. The variable *s*_*i*_ denotes the average logarithmic income of district *i*, standardized to have zero mean. Hence, the parameter θ_*j*1_ allows intramonth seasonalities to depend on district‐level income.

We are interested in the deviation—and the income gradient of the deviation—of daily purchases from their usual patterns, hence we model the random time effect as *u*_0*t*_ + *u*_1*t*_ *s*_*i*_, where *u*_0*t*_ and *u*_1*t*_ are both zero mean, serially uncorrelated, normally distributed random variables, also independent from each other and from the *ɛ*_*it*_ residuals. A high value of *u*_0*t*_ implies that purchases were unusually high on day *t*, while a high value of *u*_1*t*_ indicates that the difference between large‐ and low‐income districts was unusually high on that day. We estimate the model with maximum likelihood on data excluding February and March (the 2 months that may contain the periods of panic buying), and then predict *u*_0*t*_ and *u*_1*t*_ for the whole period.

## 5 RESULTS

**FIGURE 2:**
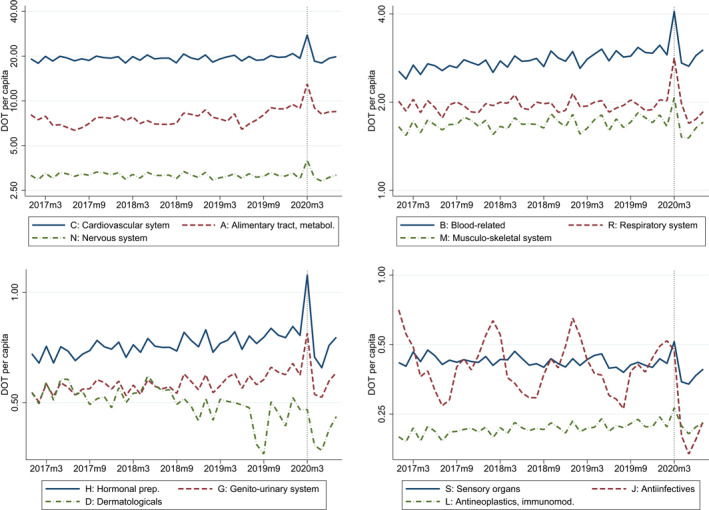
Time patterns of pharmaceutical purchases. *Note*: Gender‐ and age‐adjusted monthly DOT per capita on the logarithmic scale of the ATC1 drug categories, January 2017–July 2020

The descriptive plots of Figure 2 and the regression results of Figures A1 and A2 in the supplementary information material show that except for dermatologicals (ATC D) and anti‐infectives for systemic use (ATC J), there was a clear temporary surge in the purchases of all categories of pharmaceuticals in March 2020. The magnitude of the jump ranged between 10% (e.g., antineoplastic and immunomodulating agents, ATC L) and 40% (alimentary tract and metabolism, ATC A). A regression of total DOT (of all pharmaceuticals) would yield an overall effect of 33% for March 2020 (not shown in the figures).

**FIGURE 3:**
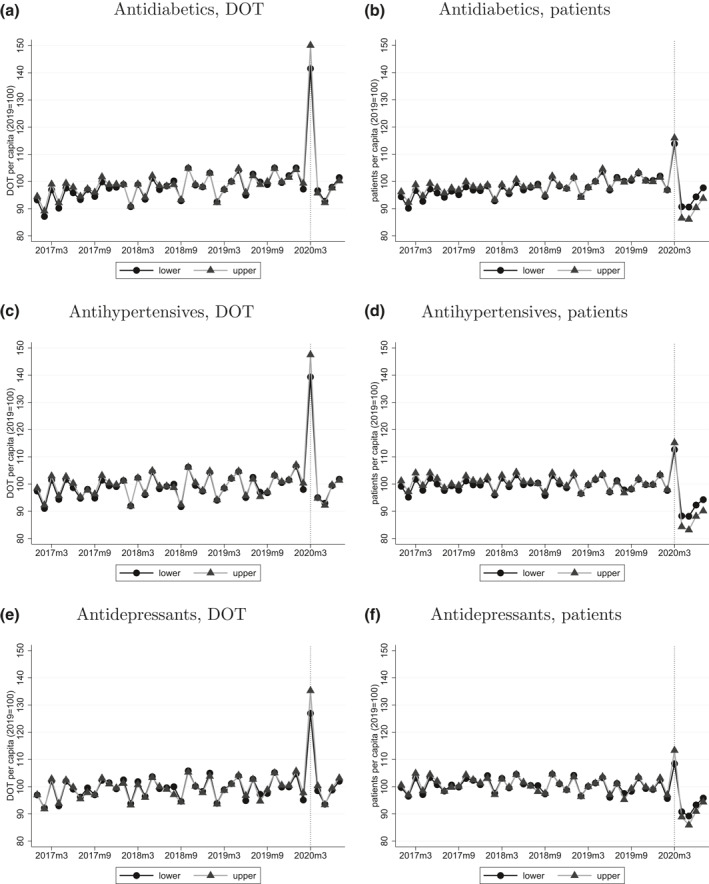
Monthly DOT and number of patients per capita by income of the district. *Note*: Gender‐ and age‐adjusted monthly DOT and number of patients per capita, normalized to 100 in year 2019, by income of the district (split at the median income), January 2017–July 2020

**FIGURE 4:**
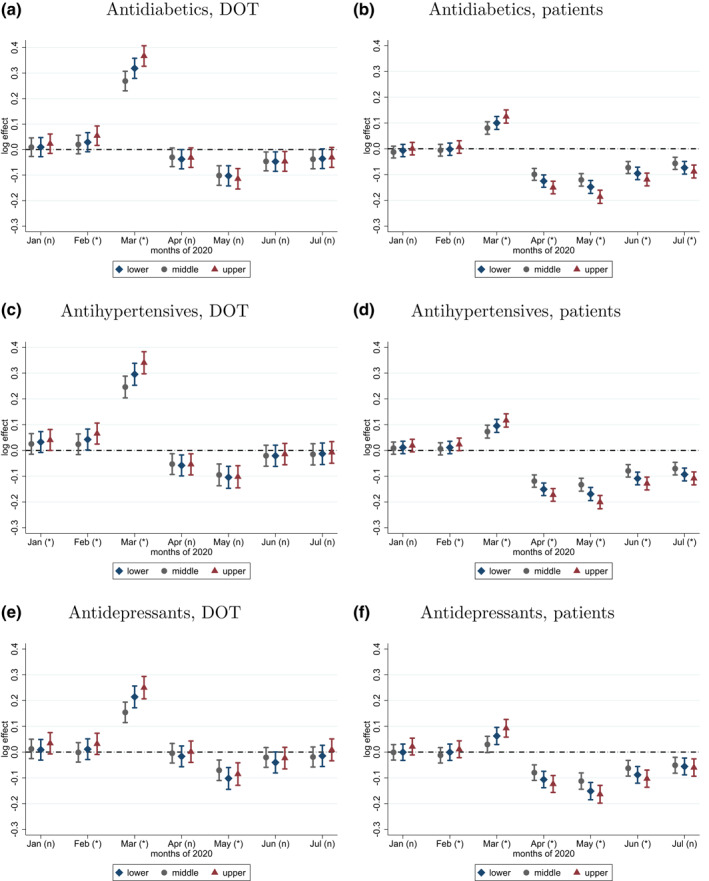
Monthly effects by income tertile on DOT and number of patients per capita. *Note*: Estimated monthly parameters (*δ*_*qk*_ in Equation 1) with 99% confidence intervals of gender‐ and age‐adjusted logarithmic DOT and number of patients per capita for three drug categories in 2020, by income tertile of the district. Heterogeneity of parameters by income tertile: (*) significant, (*n*) not significant at the 1% level

Focusing on three narrower groups of pharmaceuticals—antidiabetics, antihypertensives, and antidepressants—the descriptive and the regressions results of Figures 3 and 4 indicate that the relative surge in March 2020 was much larger for per capita DOT (20%–30%) than for the number of patients (10% or less). Also, in April–July 2020, the number of patients was well below the preshock level, while per capita DOT approached it. Hence, the amount of purchases per pharmacy visit increased from March 2020 (per capita DOT was especially low in May 2020, when it became apparent that there would be no major disruption in pharmaceutical supply, hence patients could use up the stockpiled medications.)

We also see that the relative magnitude of panic buying was larger in the richer than in the smaller districts (for DOT, by 5%–6% larger in the upper tertile and by 4%–5% smaller in the lower tertile than in the middle tertile). Actually, a more detailed regression analysis by income decile shows that purchases of antidiabetic and antihypertensive medications increased disproportionately in the uppermost decile in March (by 8%–10% more than the median), while the differences in the other deciles were more gradual (see Figure A3 in the supplementary information material). Heterogeneity by income holds for most other pharmaceutical categories as well (see Figures A1 and A2 in the supplementary information material). For the three specific pharmaceutical groups, the relative drop in the number of patients after March 2020 was also bigger in the richer districts (Figure 4).

**FIGURE 5:**
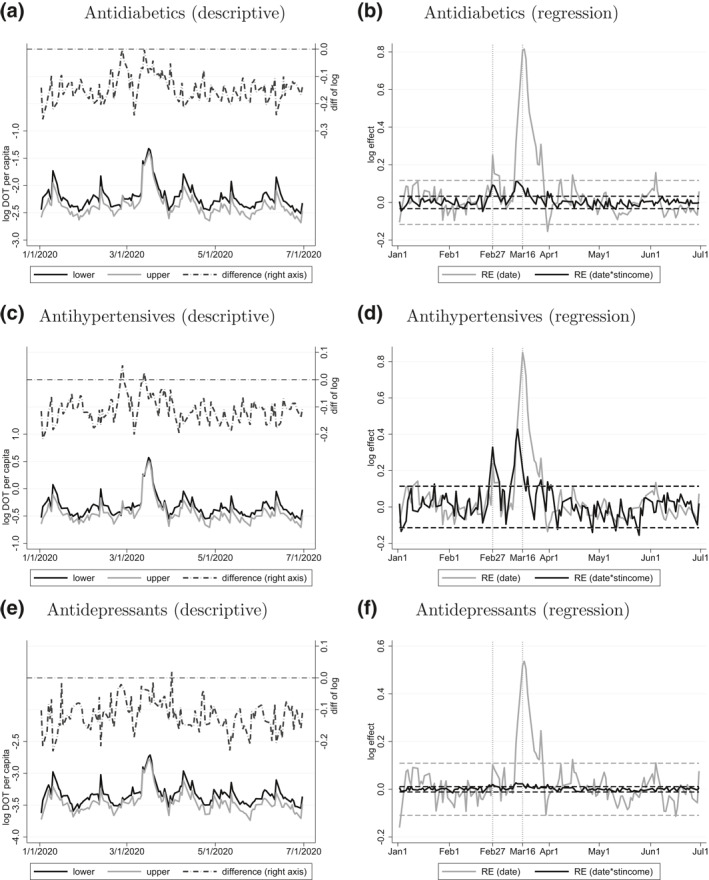
Results for DOT per capita based on daily data. *Note*: Daily logarithmic DOT per capita for three drug categories by income of the district (split at the median) and the difference by income (left column) and estimated daily random effects (*u*_0*t*_ and *u*_1*t*_ from Equation 2) of logarithmic DOT of three drug categories, with 95% prediction intervals (right column)

The results based on the daily data (Figure 5) show that in the case of antidiabetics and antihypertensives, a significant income gradient (i.e., significantly positive *u*_1*t*_ in Equation 2) appeared already at the end of February 2020, when the disease started to be considered as a major threat for Hungary (see Figure 1). We also estimate a positive income gradient on March 12–13, 2020, the days before the peak of panic‐buying (16 March), indicating that the population of richer districts responded both earlier and more to the threat of COVID‐19. Finally, the value of *u*_0*t*_ on March 16 shows that antidiabetic and antihypertensive purchases were more than twice their usual values that day.

## 6 DISCUSSION

We analyzed the timing, magnitude, and income dependence of pharmaceutical panic buying around the outbreak of the COVID‐19 pandemic in Hungary. We found that the days of therapy of pharmaceutical purchases increased by more than 30% in the month when major lockdown measures were announced. This pattern holds for almost all categories of pharmaceuticals. The estimated relative increase is in line with the international evidence on the magnitude of panic buying of pharmaceuticals (Kostev & Lauterbach, 2020) and of other goods (Baker et al., 2020; O'Connell et al., 2020). After the panic reactions, the frequency of pharmacy visits decreased and the aggregate amount of pharmaceutical purchases gradually returned to their preshock levels.

The panic buying reaction was significantly stronger in richer geographical areas, where people also reacted earlier to pandemic‐related news. While we focused on income differences in panic reactions, income can be considered as a composite indicator of socioeconomic position, access to healthcare, and access to information. Indeed, district‐level income in Hungary is strongly negatively correlated with, for example, the distance to the nearest pharmacy or with the district‐level ratio of unfilled primary care practices (Bíró et al., 2021), but strongly positively correlated with the ratio of internet subscribers in the district (based on TeIR data). We conclude that the income gradient in pharmaceutical panic buying can be driven by three mechanisms: first, by direct income effects (poorer individuals cannot stockpile pharmaceuticals due to liquidity constraints); second, by better access to pharmacies and physicians in richer districts; and third, by better access to pandemic‐related information in richer districts (as discussed in the Section 1, panic buying could be a rational reaction). While we cannot specifically test these mechanisms, it is worth noting that the income gradient of the panic reaction was similar for the three major drug categories examined, although the out‐of‐pocket cost per DOT was two to three times higher for antidiabetics than for antihypertensives and antidepressants (own calculations based on 2019 figures). This suggests that the income gradient is not solely driven by direct income effects (which would imply stronger gradient for more expensive pharmaceuticals).

Our results point out that panic buying of pharmaceuticals due to a major shock event can have detrimental effects on the vulnerable population who can react to the shock only with delays and to a smaller extent. This is particularly concerning if the panic eventually leads to temporary shortages of pharmaceuticals. Therefore, it is essential that governments prevent unnecessary stockpiling primarily with the help of appropriate communication and quantity limits.

## Supporting information

Supplementary Material

## Data Availability

The data that support the findings of this study are available from the National Health Insurance Fund Administration. Restrictions apply to the availability of these data, which were used under license for this study. Data are available from the corresponding author with the permission of the National Health Insurance Fund Administration.
