# Supplementary material for: Income gradient of pharmaceutical panic buying at the outbreak of the COVID‐19 pandemic
Source: Health Econ. 2021 Jul 3;30(9):2312–20. doi: 10.1002/hec.4378 (PMC8420393; doi:10.1002/hec.4378)
Supplement: Supplementary file 1 — Supplementary Material [file HEC-30--s001.pdf]

Appendix

(a) A: Alimentary tract and metabolism

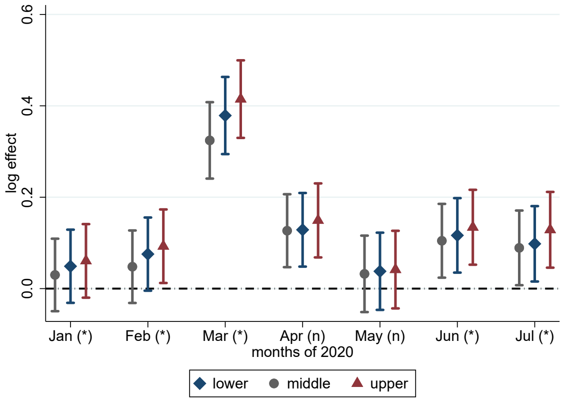

(b) B: Blood and blood forming organs

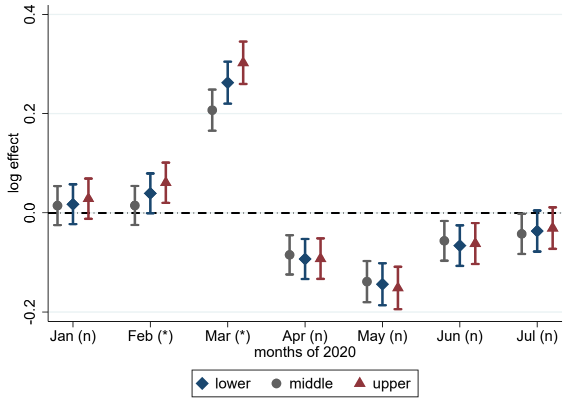

(c) C: Cardiovascular system

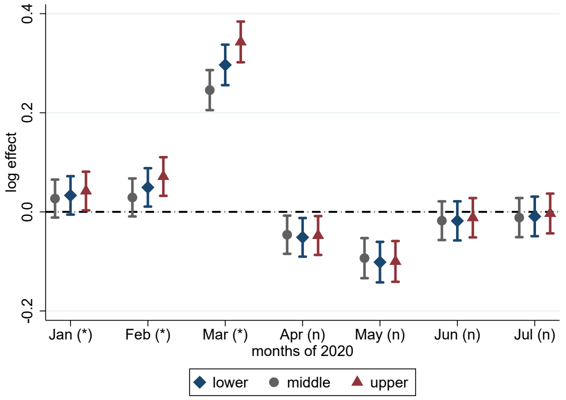

(d) M: Musculo-skeletal system

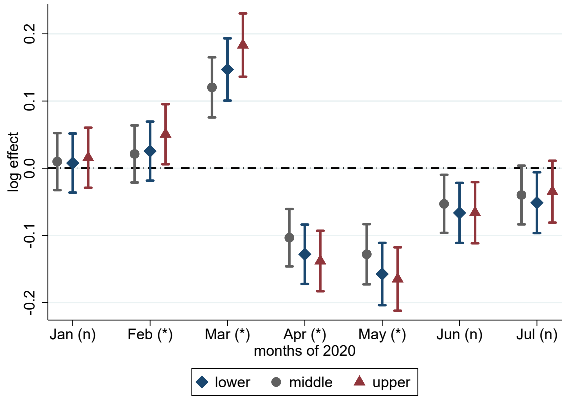

(e) N: Nervous system

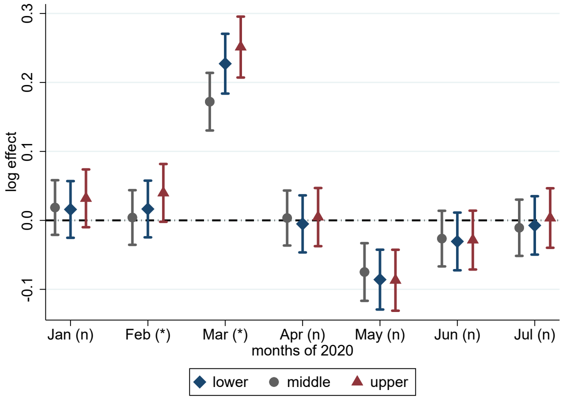

(f) R: Respiratory system

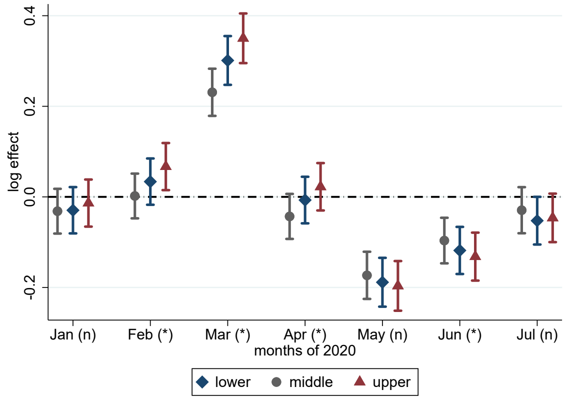

Note: Estimated monthly parameters (δ_{qk} in equation (1)) with 99% confidence intervals of gender- and age-adjusted logarithmic DOT per capita for the largest ATC1 categories in 2020, by income tertile of the district. Heterogeneity of parameters by income tertile: (*) significant, (n) not significant at the 1% level.

Appendix Figure A1: Largest ATC1 categories: monthly effects by income tertile on DOT per capita

(a) D: Dermatologicals

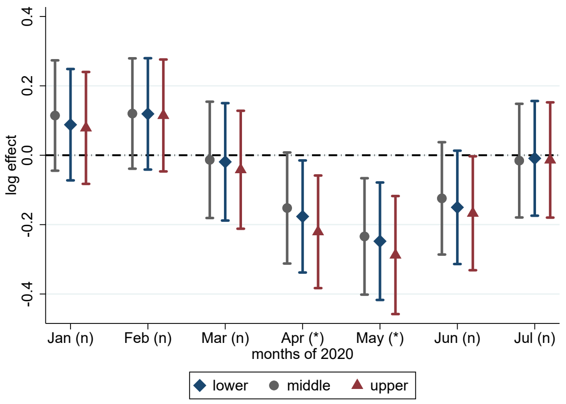

(b) G: Genito-urinary system

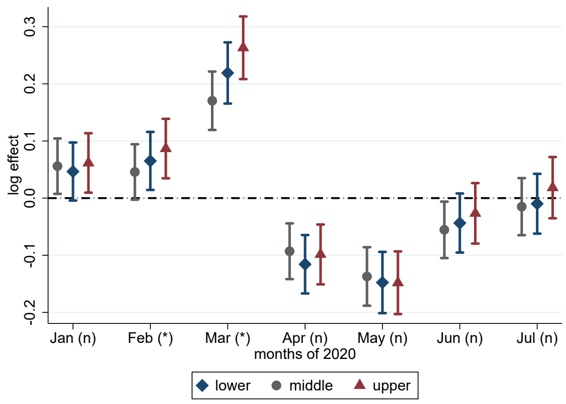

(c) H: Hormonal preparations

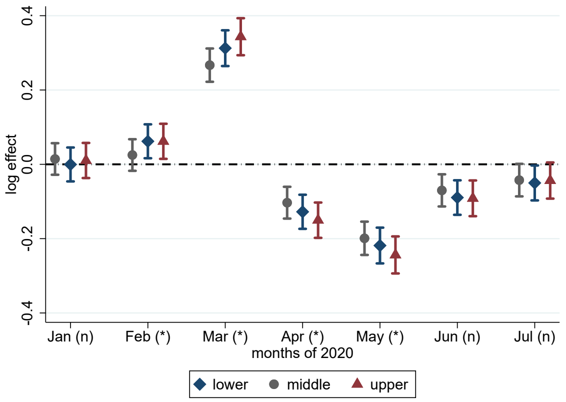

(d) J: Antiinfectives

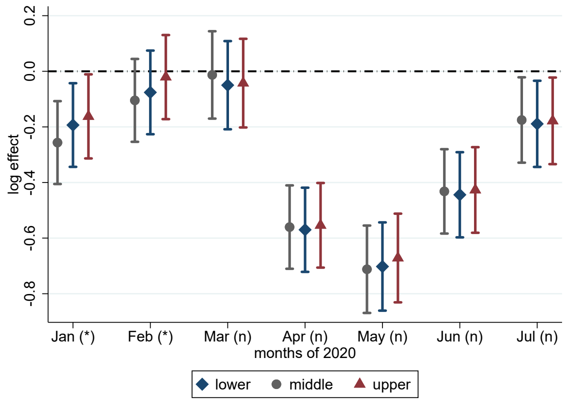

(e) L: Antineoplastics, immunomodulation

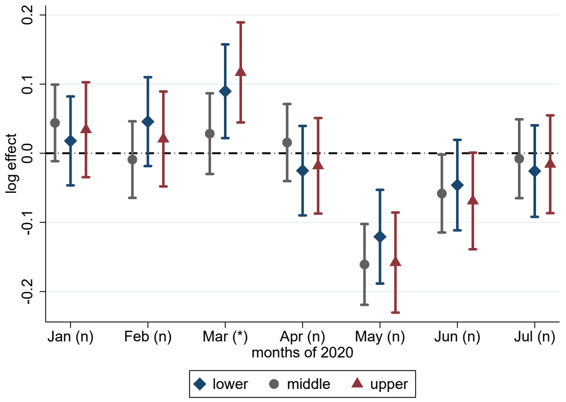

(f) S: Sensory organs

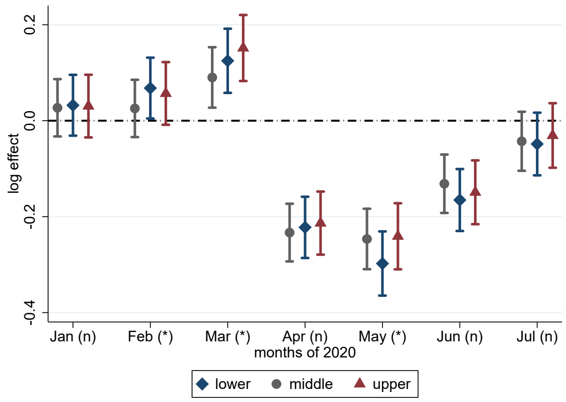

Note: Estimated monthly parameters (δ_{qk} in equation (1)) with 99% confidence intervals of gender- and age-adjusted logarithmic DOT per capita for further ATC1 categories in 2020, by income tertile of the district. Heterogeneity of parameters by income tertile: (*) significant, (n) not significant at the 1% level.

Appendix Figure A2: Further ATC1 categories: monthly effects by income tertile on DOT per capita

(a) Antidiabetics

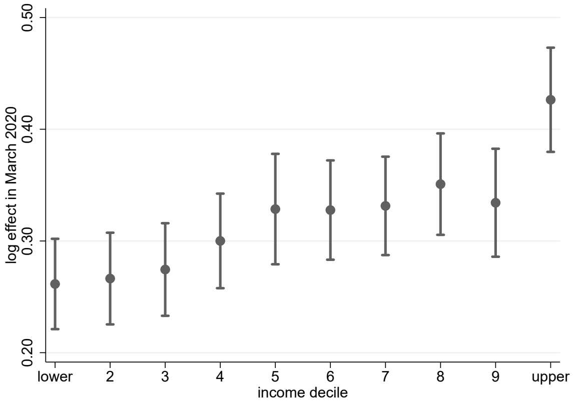

(b) Antihypertensives

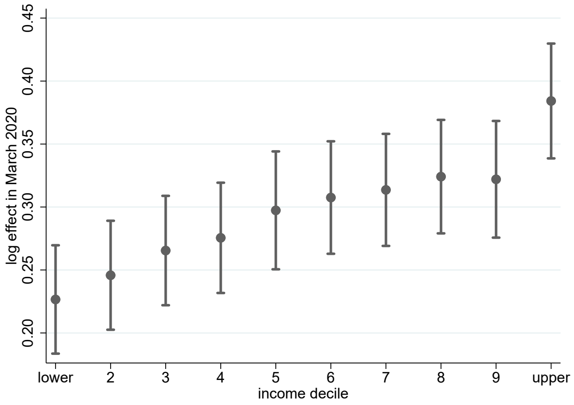

(c) Antidepressants

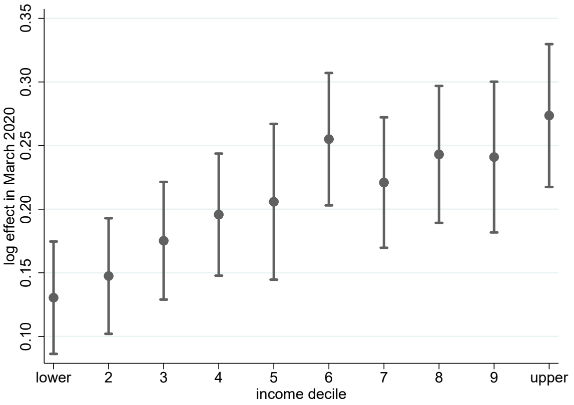

Note: Estimated decile-specific parameters (δ_{qk} in equation (1), with q indexing income deciles here) for March 2020 with 99% confidence intervals of gender- and age-adjusted logarithmic DOT for three drug categories.

Appendix Figure A3: Effects for March 2020 by income decile
